# Defects in Innate Immunity Render Breast Cancer Initiating Cells Permissive to Oncolytic Adenovirus

**DOI:** 10.1371/journal.pone.0013859

**Published:** 2010-11-05

**Authors:** Laura Ahtiainen, Cristina Mirantes, Tiina Jahkola, Sophie Escutenaire, Iulia Diaconu, Pamela Österlund, Anna Kanerva, Vincenzo Cerullo, Akseli Hemminki

**Affiliations:** 1 Transplantation Laboratory, Cancer Gene Therapy Group, Molecular Cancer Biology Program, Haartman Institute and Finnish Institute of Molecular Medicine, University of Helsinki, Helsinki, Finland; 2 HUSLAB, Helsinki University Central Hospital, Helsinki, Finland; 3 Department of Plastic Surgery, Helsinki University Central Hospital, Helsinki, Finland; 4 Viral Infections Unit, Department of Vaccination and Immune Protection, National Institute for Health and Welfare, Helsinki, Finland; 5 Department of Obstetrics and Gynaecology, Helsinki University Central Hospital, Helsinki, Finland; The University of Chicago, United States of America

## Abstract

**Background:**

Cancer stem cells/initiating cells (CSC/CIC), are thought to exist as a small population in malignant tissues. They are resistant to conventional cancer treatments and possibly underlie post-treatment relapse. The CIC population can be targeted with capsid modified oncolytic adenoviruses.

**Methodology/Principal Findings:**

We studied the mechanisms of innate immunity to oncolytic adenovirus Ad5/3-Delta24 in conventional treatment resistant non-CIC breast cancer cells, breast cancer CD44^+^/CD24^−/low^ CIC population and normal breast tissue CD44^+^/CD24^−/low^ stem cells. We compared virus recognition by pattern recognition receptors for adenovirus, Toll-like receptors (TLR) 2 and 9 and virus induced type I interferon (IFN) response regulation in these cell types. We show TLR mediated virus recognition in these non-immune cell types. Normal tissue stem cells have intact type I IFN signaling. Furthermore, TLR9 and TLR2 reside constantly in recognition sites, implying constant activation. In contrast, breast cancer CD44^+^/CD24^−/low^ CIC have dysregulated innate immune responses featuring dysfunctional virus recognition caused by impaired trafficking of TLR9 and cofactor MyD88 and the absence of TLR2, having a deleterious impact on TLR pattern recognition receptor signaling. Furthermore, the CIC have increased inhibitory signaling via the suppressor of cytokine signaling/Tyro3/Axl/Mer receptor tyrosine kinase (SOCS/TAM) pathway. These defects in contribute to dysfunctional induction of type I IFN response in CIC and therefore permissivity to oncolytic adenovirus.

**Conclusions/Significance:**

CICs may underlie the incurable nature of relapsed or metastatic cancers and are therefore an important target regarding diagnostic and prognostic aspects as well as treatment of the disease. This study addresses the mechanisms of innate infection immunity in stem cells deepening the understanding of stem cell biology and may benefit not only virotherapy but also immunotherapy in general.

## Introduction

Several cancer types have been shown to contain a small population of cells that are resistant to conventional treatments and may contribute to post-treatment relapse. This population possesses a greater ability to maintain tumor formation than other tumor cell types and shares metabolic properties and markers with normal tissue specific stem cells. Therefore, these cells have been proposed to be cancer initiating cells or so called cancer stem cells (CIC/CSC). Normal stem cells allow the maintenance, regeneration and growth of adult tissues and sustain a pool of undifferentiated tissue-specific cells under regulation of local and systemic signals. In contrast, CICs have lost this control [1]. Populations with CIC properties have been identified in cancers of the hematopoietic system, brain, breast, ovary and prostate [2], [3]. In breast cancer, the CIC population has been shown to lie in the CD44^+^/CD24^−/low^ portion [2], [4], [5]. The phenotypic properties associated with CIC are slow cellular replication, the capacity for expelling anti-tumor drugs and apoptosis resistance [6]. These characteristics render them resistant to many conventional cancer therapies [4], [6], [7], [8]. CICs may underlie the incurable nature of relapsed or metastatic cancers and are therefore an important target regarding diagnostic and prognostic aspects as well as treatment of the disease [9], [10].

Capsid modified oncolytic adenoviruses can be utilized to specifically target the CD44^+^/CD24^−/low^ population [11], [12], [13], [14]. Infection of tumor cells results in selective replication, oncolysis, and subsequent release of the virus progeny through vasculature into metastases. Normal tissue is spared due to engineered alterations in the virus genome [12]. These viruses are emerging as novel tools for cancer therapy and several are already in clinical trials [15], [16]. Virotherapy can also be utilized to sensitize tumor cells to radiation and chemotherapy and also as tools for immunotherapy [17]. Thus, oncolytic viruses have significant advantages for improving treatment options for patients [18].

The innate immune responses, mediated by type I interferons (IFN) and cytokines such as IL6 and TNF-α, are key in defining permissivity of different cell types to viral infection. These phenomena are well characterized in immune cells whereas still poorly understood in the context of stem cells. Normal tissue stem cells display resistance to viral infection whereas the CIC containing population appears permissive to viral infection by oncolytic viruses [11], [19], [20], [21]. Several cytokines modulate CIC functions and understanding these interactions is central in targeting the cancer cell population [22]. Variable defects in interferon response may confer cancer cells a growth or survival advantage. However, dysfunction in interferon production or responsiveness to interferons results in a compromised antiviral response.

Virus-induced type I IFN response is mediated by type I IFN receptor (IFNAR), interferon regulatory transcription factors (IRFs) and transcription factor NFκB. A second signaling pathway involves Toll-like receptors (TLRs) that are expressed on the cell surface or in endosomes. TLRs signal through adaptor proteins including MyD88, TRIF and TRAF6 [23] activating signal cascades employing the MAP kinase, NFκB and IRF. The main pathogen pattern recognition receptors (PPRs) for adenoviruses are TLR9 in the endosomes and TLR2 on the cell surface [24], [25]. Both signal MyD88-dependently activating NF-κB and MAPK cascades leading to the production of type I IFNs inducing an antiviral state[26]. The different routes of activation depend on the cell type. To limit potentially harmful inflammation, responses are attenuated by Tyro3/Axl/Mer (TAM) family of receptor tyrosine kinases. TAM receptors act in conjunction with the IFNAR/STAT signaling cassette driving the expression of suppressor of cytokine signaling (SOCS) proteins that inhibit signaling via STAT and TLR pathways.

Previous data suggested that CD44^+^/CD24^−/low^ CIC populations are more permissive to Ad5/3-Delta24 capsid modified oncolytic adenovirus, than the cell population in the corresponding normal tissue, and can be killed by oncolytic virus [11], [13], [21]. We have here studied whether this is caused by dysregulation of innate immune responses. We explore innate immunity pathways in breast cancer CIC and compare them to non-CIC but treatment resistant breast cancer cells and normal mammary tissue cells. This study broadens the understanding of innate immunity in normal breast stem cells and the cancer initiating population. Modulation of these pathways may improve viral oncolytic efficacy in cancer stem cell targeting cancer therapy.

## Results

### Characterization of cell populations in normal breast tissue derived mammospheres and breast cancer patient pleural effusion explant derived JIMT-1 and ArLa

Cells isolated from normal mammary tissue cultured *in vitro* and two conventional cancer treatment resistant breast cancer patient pleural effusion explant derived cell lines, JIMT-1 and ArLa, were analyzed for phenotypic properties. We have previously shown that JIMT-1 CD44^+^/CD24^−/low^ reconstitute tumors *in vivo* in a xenograft mouse model [11]. Here we further analyzed universal undifferentiated stem cell markers and CD44 and CD24 status to identify proportions of prospective normal tissue stem cell/CIC populations.

**Figure 1:**
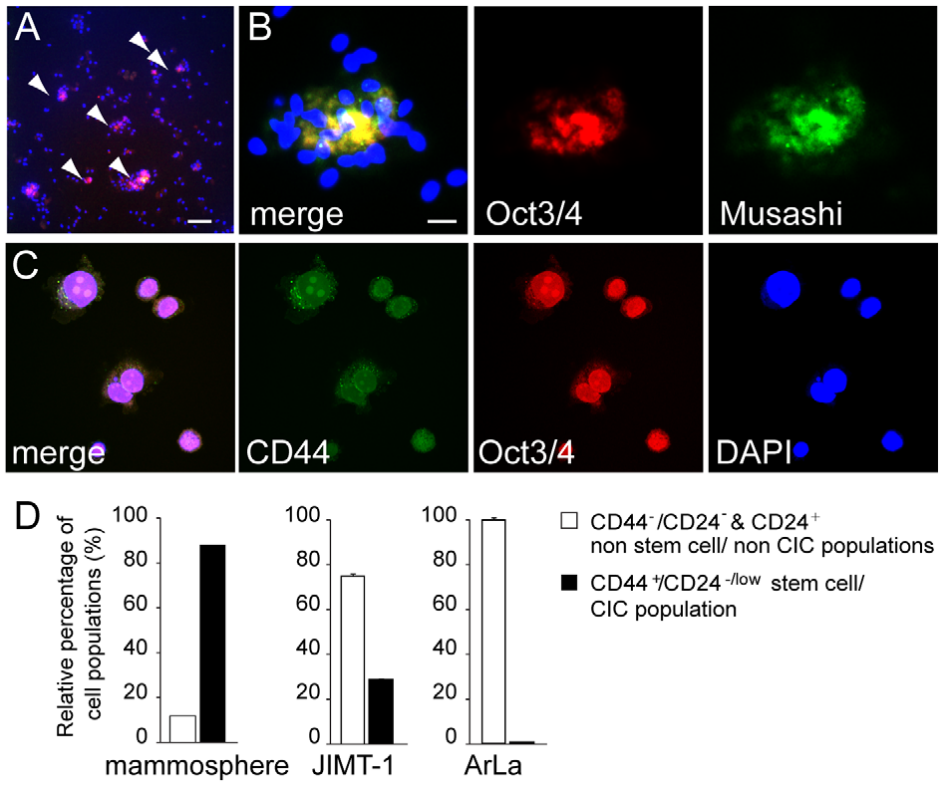
Characterization of normal breast tissue mammosphere, JIMT-1 and ArLa breast cancer patient pleural effusion explant derived cell line stem cell populations. To characterize the distribution of prospective stem cell/cancer stem cell populations normal breast tissue derived mammospheres grown in stem cell enriching conditions (A, arrowheads) and JIMT-1 breast cancer patient pleural effusion explant derived cell line were immunostained with a undifferentiated stem cell marker Oct3/4 and mammary stem cell marker Musashi, nucleai stained with DAPI (blue, A–C). (A and B) Mammospheres show positivity for Oct3/4 (red) and Mushashi (green). (C) JIMT-1 breast cancer patient pleural effusion explant derived cell line similarly shows positivity for Oct3/4 (red) and also CD44+ (green). Scale bars: A 80 µm, B and C 20 µm (D) Distributions of non-cancer initiating (non-CIC) populations CD44^−^/CD24^−^ and CD24+ and CIC/stem cell containing CD44^+^/CD24^−/low^ population in mammospheres, JIMT-1 and ArLa breast cancer cell lines (D).

The cells isolated from normal mammary tissue formed self-renewing spheroids in attachment independent conditions in the absence of serum (Fig. 1A). These spheres could be passaged up to four times and showed positivity for the undifferentiated stem cell marker Oct3/4 and mammary stem cell marker Musashi (Figs. 1A and B). Similarly, the JIMT-1 breast cancer patient pleural effusion explant derived cells were positive for CD44 and Oct3/4 respectively (Fig. 1C). Mammospheres were enriched with of CD44^+^/CD24^-/low^ population containing 87% of this cell type (Fig. 1D). The JIMT-1 were also enriched in CD44^+^/CD24^−/low^ containing 25% of the prospective CIC-population (Fig. 1D). In contrast, the ArLa contained less than 1% CD44^+^/CD24^−/low^ cells (Fig. 1D) and were thus defined as the non-initiating (non-CIC) cancer cell type.

### Endogenous TLR9 resides persistently in endosomes in CD44^+^/CD24^−/low^ normal mammary tissue cells

TLR9 and TLR2 are specific pathogen recognition receptors of adenovirus [24], [25]. The localization of the innate immune sensors at the cellular level is of key importance for their physiological function and any misplacement may result in an impaired response. We hypothesized that mislocalization of these receptors in mammary tissue CD44^+^/CD24^−/low^ stem cells and/or CIC can result in an impaired immune response. Therefore, we analyzed the localization of endogenous TLR9, TLR2 and cofactor MyD88 by immunofluorescence staining and confocal microscopy in sorted non-infected cells and cells sorted and infected with a chimeric oncolytic adenovirus bearing the knob domain of the adenovirus serotype 3 (Ad5/3-Delta24).

**Figure 2:**
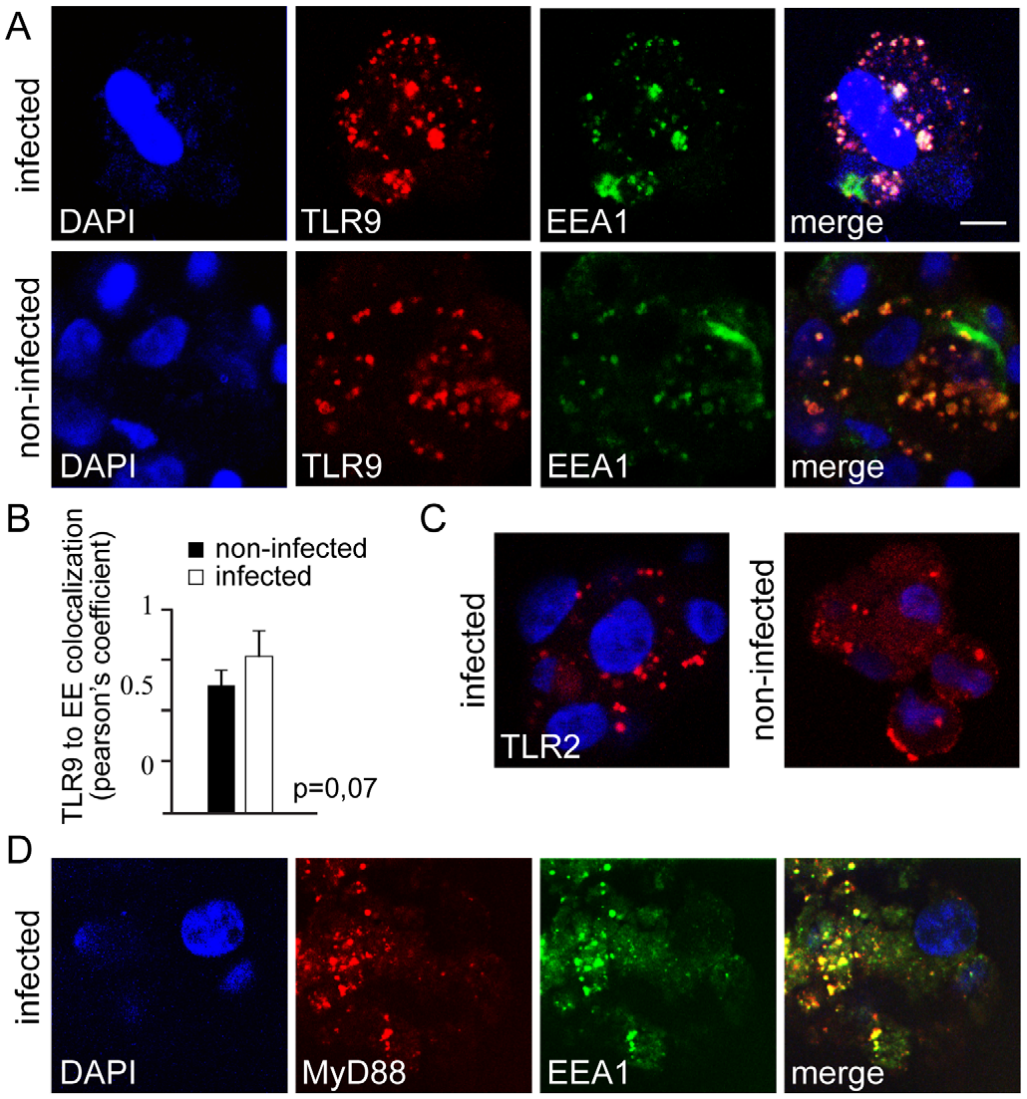
TLR9 and TLR2 reside constantly in sites of virus recognition in normal breast tissue stem cells. (A) Immunofluorescence staining of endogenous TLR9 (red) in normal mammary stem cells shows colocalization with an endosomal marker EEA1 (green) in both oncolytic adenovirus Ad5/3-Delta24 infected cells at 6 h after infection and also non-infected cells (A), DAPI nuclear staining in blue. (B) Quantization of colocalization: The graph represents Pearson's coefficient of TLR9 and endosome (EE) colocalization from three independent fields of cells in two different experiments. Colocalization of TLR9 to endosomal marker EEA1 (EE) shows no difference between infected and non-infected normal stem cells (error bars SD, p = 0.07) (B). Endogenous TLR2 staining (red) in normal stem cells shows cell surface and partly endosomal distribution in both infected and non-infected cells (C). Endogenous MyD88 (red), a cofactor in TLR signaling, is present in infected cells and associates with endosomal structures (green), DAPI in blue (D). The distributions of the pathogen pattern recognition receptors in mammospheres suggest constant activation of the receptors in this cell type (C). Scale bar:10 µm.

In normal mammary tissue CD44^+^/CD24^−/low^ stem cells TLR9 localized in endosomes in Ad5/3-Delta24 infected cells, as indicated by colocalization with an endosomal marker EEA1 and persisted still in the endosomes 6 h after infection (Figs. 2A, B). Interestingly TLR9 and TLR2 were found in the endosomes also in uninfected normal mammary tissue CD44^+^/CD24^−/low^ stem cells cells (Figs. 2A-C) suggesting constant activation of the receptors in this cell type. Colocalization analysis of TLR9 and EEA1 in showed a similar pattern in both infected and non-infected cells (Fig. 2B). The cofactor of TLR2 and TLR9 signaling MyD88 was also found to be associated with endosomes in infected CD44^+^/CD24^−/low^ normal mammary tissue cells (Fig. 2D).

### Virus recognition by TLR9 and TLR2 in ArLa non-CIC and mislocalization of endogenous TLR9 and absence of endogenous TLR2 in JIMT-1 CD44^+^/CD^24−/low^ CIC

**Figure 3:**
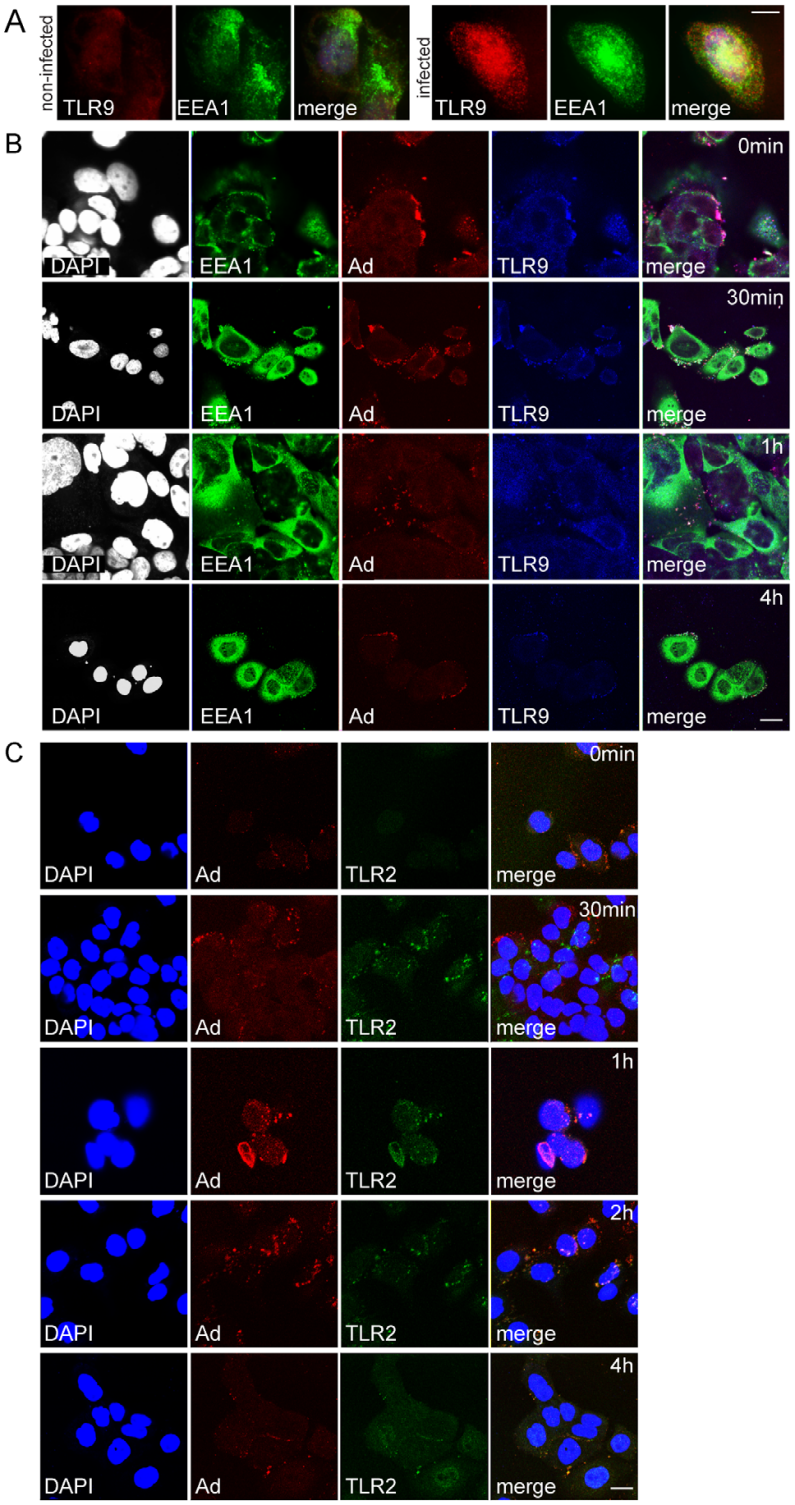
Oncolytic adenovirus recognition by TLR9 and TLR2 in ArLa non-CIC breast cancer cells. Immunofluorescence staining of TLRs in non-CIC ArLa breast cancer cells: (A) In non-infected non-CIC ArLa low level of TLR9 staining (red) is present and upon infection with Ad5/3-Delta24 oncolytic adenovirus TLR9 is upregulated and the localization becomes endosomal, as indicated by colocalization with endosomal marker EEA1 (green), indicating normal trafficking of the receptor. (B) Staining of adenovirus hexon (red) in infected ArLa cells at 0 min, 30 min, 1 h and 4 h time points following infection, shows initial localization of the virus on cell surfaces and at later time points at 30 min up to 4 h internalization in endosomes (EEA1, green). Nuclear counterstain (DAPI) in white. (B) Furthermore, adenovirus staining (red) colocalizes with TLR9 (blue) in endosomes (green). (C) Adenovirus staining (red) also colocalizes with TLR2 (green) in ArLa cells (DAPI in blue). These data indicate that in this non-CIC conventional cancer treatment resistant breast cancer cell type TLR9 and TLR2 are active in oncolytic adenovirus recognition. Scale bars: 10 µm.

**Figure 4:**
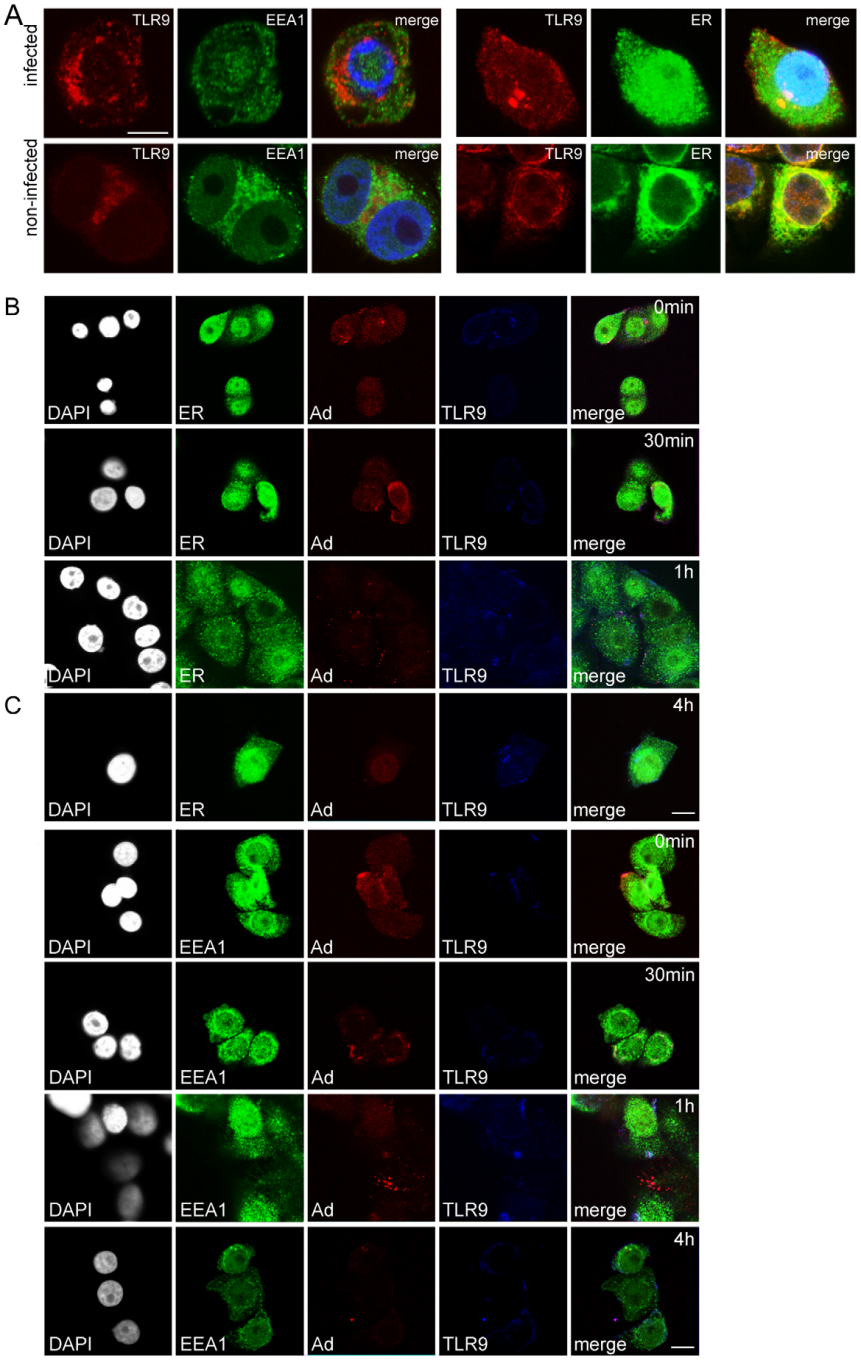
Mislocalization of TLR9 in JIMT-1 CD44^+^/CD24^−/low^ CIC breast cancer cells. In contrast to normal tissue stem cells and ArLa non-CIC cancer cells, TLR9 (red) shows mislocalization in JIMT-1 CD44^+^/CD24^−/low^ CIC population indicated by distinct distribution form the endosomal marker EEA1 (green) and partial co-localization with an ER-marker calnexin (ER, green) in both infected or non-infected cells, nuclear stain DAPI in blue (A). (B and C) Staining of adenovirus hexon (red) at 0 min, 30 min, 1 h and 4 h time points following infection, shows initial localization of the virus on cell surfaces and at later time points at 30 min up to 4 h internalization in endosomes, showing that Ad5/3-Delta24 is able to infect this cell type. DAPI nuclear staining in white (B and C). In infected JIMT-1 CD44^+^/CD24^−/low^ CIC TLR9 (blue) is retained in the ER-golgi like structures (B, green) and does not traffic to the endosomes (C, green) to colocalize with the virus at time points up to 4 h post infection (B). Scale bars:10 µm.

Endogenous TLR9 and TLR2 were next studied in Ad5/3-Delta24 infected and non-infected non-CIC ArLa cells and JIMT-1 CD44^+^/CD24^−/low^ CIC by immunofluorescence staining and confocal microscopy. Staining of adenovirus hexon showed that the virus could infect both CD44^+^/CD24^−/low^ JIMT-1 and ArLa, as positive staining was initially seen on cell surfaces and at later time points at 30 min up to 4 h internalized in endosomes (Figs. 3A-C and 4A-C). In non-infected non-CIC ArLa cell low level of TLR9 expression was seen localized in the ER and upon infection TLR9 was upregulated and localized in endosomes, indicating normal trafficking of the protein (Fig. 3A). Also, adenovirus staining colocalized with TLR9 in endosomes (Fig. 3B) and with TLR2 on the cell surface (Fig. 3C) indicating that in this breast cancer cell type TLR9 and TLR2 are active in virus recognition.

**Figure 5:**
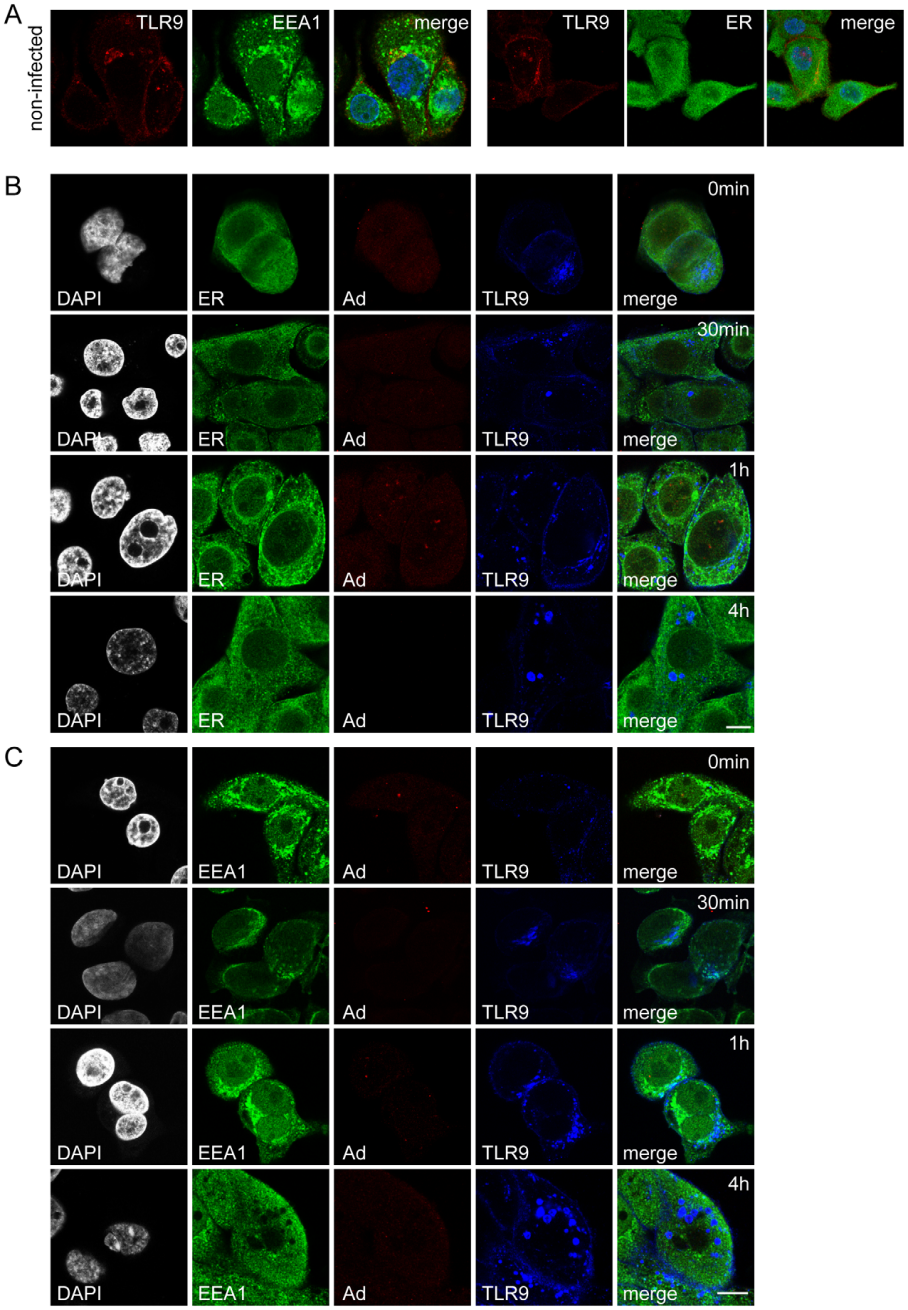
Normal trafficking of TLR9 in JIMT-1 CD44^−^/CD24^−^ non-CIC population. Immunofluorescence staining of TLR9 in JIMT-1 CD44^−^/CD24^−^ non-CIC population. (A) In non-infected non-CIC cells TLR9 (red) resides partially in endosomes and ER (EEA1 and ER, both in green). (B and C) Adenovirus staining (red) in infected JIMT-1 non-CIC population at 0 min, 30 min, 1 h and 4 h time points following infection, shows the virus on cell surfaces and internalized in endosomes (EEA1, green) together with TLR9 (blue). TLR9 is upregulated upon infection and shows intact trafficking from the ER to the endosomal compartment (C). Nuclear counterstain (DAPI) in white. These data indicate that, in contrast to the cancer initiating population of JIMT-1, the non-CIC CD44^−^/CD24^−^ cell types show intact trafficking and adenovirus recognition by TLR9. Scale bars: 10 µm.

**Figure 6:**
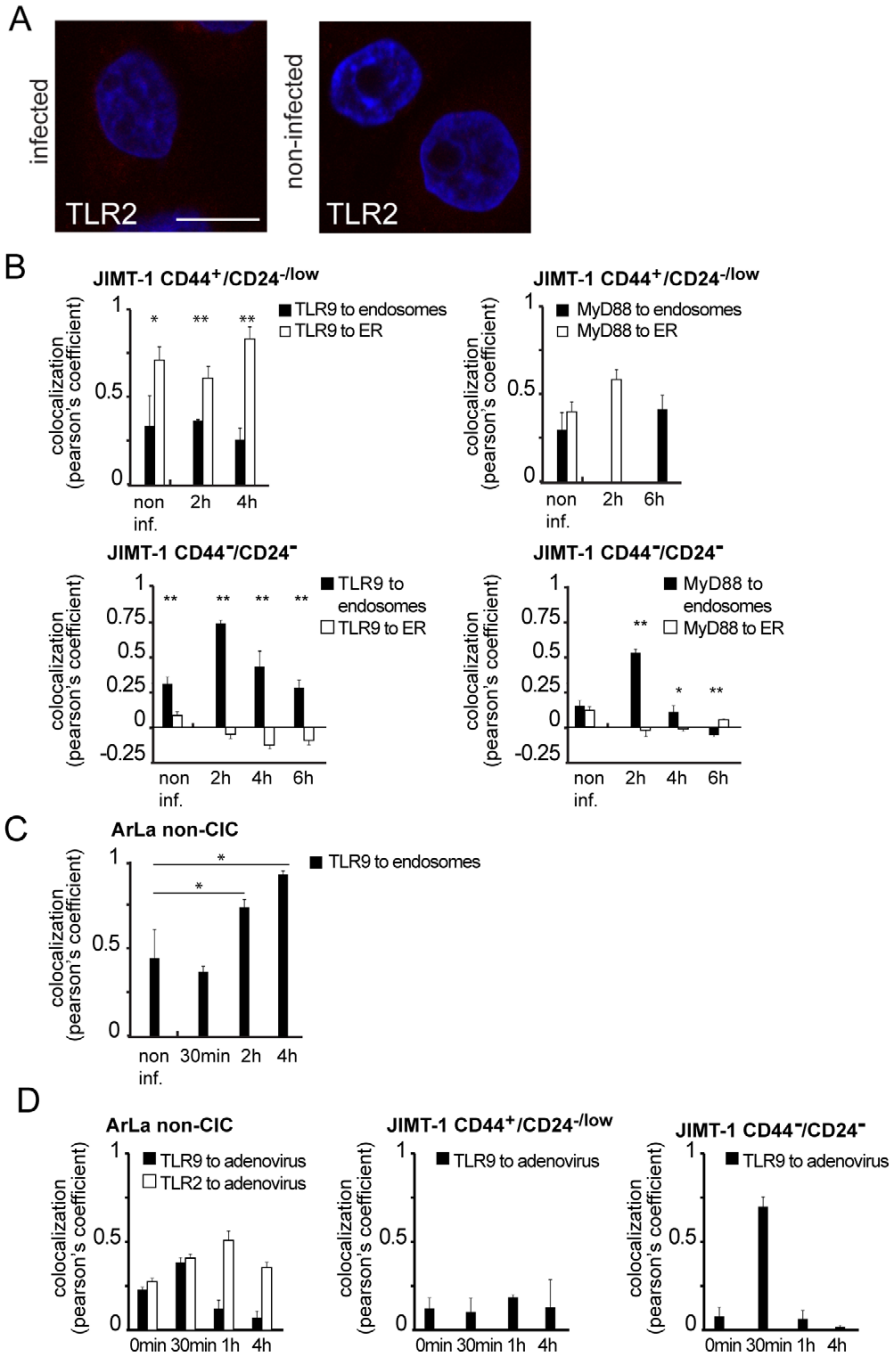
Absence of TLR2 and dysregulated trafficking of TLR9 in JIMT-1 CD44^+^/CD24^−/low^ CIC breast cancer cells. (A) Immunofluorescence staining of TLR2 (red) in JIMT-1 CD44^+^/CD24^−/low^ CIC population shows absence of the receptor in non-infected and infected cells, DAPI nuclear staining in blue. (B) Colocalization analysis of organelle markers to TLR9 and MyD88 in JIMT-1 CD44^+^/CD24^−/low^ CIC and JIMT-1 CD44^−^/CD24^−^ non-CIC cell populations. Graphs represent Pearson's coefficient of TLR9 and MyD88 colocalization to ER or endosomes (error bars: SEM). In JIMT-1 CD44^+^/CD24^−/low^ CIC TLR9 does not reach endosomes but remains colocalized with the ER up to four hours. Similarly, MyD88 does not associate with endosomes at 6 h time point (C) indicating dysfunctional trafficking of the proteins. However, in JIMT- 1 CD44^−^/CD24^−^ non-CIC cell population both TLR9 and MyD88 associate with endosomes two hours following infection. Colocalization analysis of TLR9 to endosomal marker in ArLa non-CIC shows partial colocalization at two hours and full colocalization at four hours after infection (C). Colocalization analysis of TLR9 and TLR2 to adenovirus in ArLa non-CIC verifies the recognition of adenovirus by TLR9 and TLR2 in this cell type (D). In contrast TLR9 in JIMT-1 CD44^+^/CD24^−/low^ CIC does not colocalize with adenovirus staining, whereas in CD44^−^/CD24^−^ non-CIC population TLR9 colocalizes with the adenovirus staining similarly to ArLa non-CIC (D).

In non-infected JIMT-1 CD44^+^/CD24^−/low^ low levels of TLR9 were present in the ER (Fig. 4A). However, in contrast to normal tissue CD44^+^/CD24^−/low^ stem cells and ArLa non-CIC, in infected JIMT-1 CD44^+^/CD24^−/low^ CICs endogenous TLR9 was mislocalized in ER-Golgi like structures and not in endosomes (Fig. 4A and B) and also did not colocalize with adenovirus in the endosomes (Fig. 4B and C) indicating dysregulated trafficking and dysfunctional virus recognition by TLR9. The trafficking of TLR9 in JIMT-1 CD44^-^/CD24^-^ non-CIC population showed a similar pattern as ArLa non-CIC: The receptor was found in the ER and at low levels in endosomes in non-infected cells (Fig. 5A) and upon infection TLR9 was upregulated and trafficked from the ER to the endosomes (Fig. 5 B and C). In contrast to normal stem cells and ArLa non-CIC cells, TLR2 was undetectable or severely reduced in non-infected and infected JIMT-1 CD44^+^/CD24^−/low^ CIC (Fig. 6A).

Colocalization analysis of TLR9 and MyD88 to ER or endosomes in JIMT-1 CD44^+^/CD24^−/low^ CICs showed that neither TLR9 nor MyD88 fully reaches endosomes in 4 h and 6 h respectively (Fig. 6B), whereas in the CD44^−^/CD24^−^ non-CIC population localization became endosomal upon virus infection (Fig. 6B). Colocalization analysis of TLR9 to endosomal marker EEA1 in infected ArLa non-CIC showed that TLR9 partially colocalizes with endosomes at two hours and fully four hours after infection (Fig. 6C). Colocalization analysis of TLR9 and TLR2 to adenovirus in ArLa non-CIC verifies the recognition of adenovirus by TLR9 and TLR2 in this cell type at respective time points (Fig. 6D). In contrast TLR9 in JIMT-1 CD44^+^/CD24^−/low^ CIC does not colocalize with adenovirus staining (Fig. 6 D). In JIMT-1 CD44^−^/CD24^−^ non-CIC population the localization pattern of TLR9 in respect to adenovirus was similar to non-CIC ArLa (Fig. 6D). Defective trafficking of TLR9 and MyD88 in JIMT-1 CD44^+^/CD24^−/low^ CIC was further confirmed by transient transfection of constructs expressing fluorescently labeled proteins and organelle markers with live cell imaging (Supplementary Fig. S1).

In conclusion, non-CIC breast cancer cells feature oncolytic adenovirus recognition by TLR9 and TLR2 and intact TLR trafficking, whereas CIC have defects in trafficking of TLR9 and co-factor MyD88 and lack of TLR2. These defects are likely to have an impact on the proper function on pathogen recognition receptor signaling impairing innate immune responses in these cells.

### Defective IFN production in ArLa non-CIC and JIMT-1 CD44^+^/24^−/low^ CIC but not CD44^+^/24^−/low^ normal breast tissue cells

As innate immune recognition of the oncolytic adenovirus seemed to be compromised in CIC we next assayed type I IFN production in response to virus infection by quantitative real time PCR in CD44^+^/CD24^−/low^ normal stem cells, ArLa non-CIC cells and JIMT-1 CD44^+^/CD24^−/low^ CICs. Cells were sorted and infected with Ad5/3-Delta24 and four and 24 hours following infection, mRNA was collected and analyzed for type I interferons (IFNα and IFNβ) interferon regulatory factors IRF3, IRF7 and transcription factor STAT1 expression.

**Figure 7:**
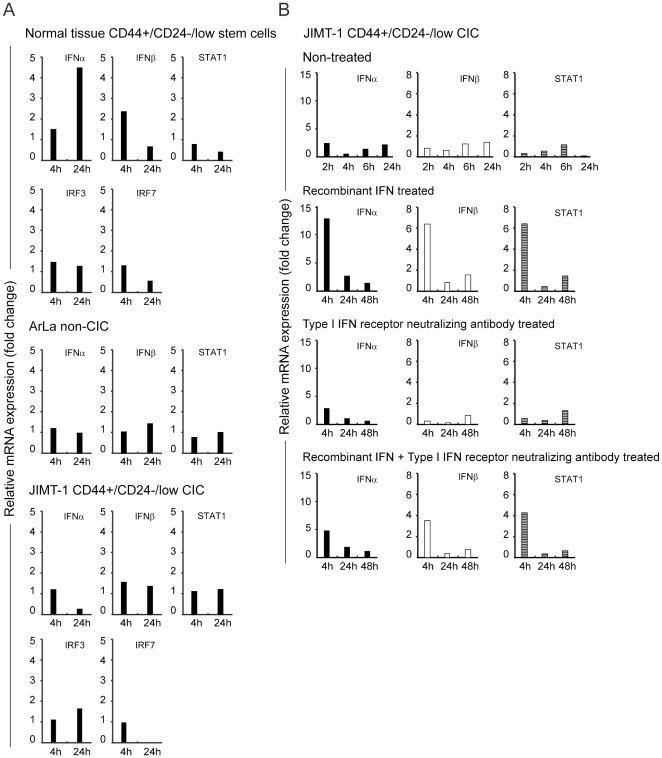
Intact type I IFN response in normal stem cells and defective type I IFN response in JIMT-1 CD44^+^/CD24^−/low^ CIC and ArLa non-CIC breast cancer cells. (A) Normal breast tissue derived mammospheres, ArLa non-CIC breast cancer cells and JIMT-1 CD44^+^/CD24^−/low^ CIC and were infected with an oncolytic adenovirus Ad5/3-Delta24 and total cellular RNA was collected at 4 h and 24 h after infection. RNA was isolated for cDNA synthesis, and semiquantitative RT-PCR was performed to determine relative IFNα, IFNβ, IRF3, IRF7 and STAT1 mRNA levels. Data are representative of three individual experiments. (B) Type I IFN induction in JIMT-1 CD44^+^/CD24^−/low^ CIC treated with recombinant universal type I interferon and/or with interferon receptor neutralizing antibody and relative mRNA levels assayed by qPCR: Exogenous IFN results in induction of IFNα, IFNβ and STAT1 at 4 h and the levels are downregulated to baseline at 24 h. Induction is inhibited by receptor neutralizing antibody. These results imply that the defect in innate immune response in CD44^+^/CD24^−/low^ CIC is caused at least partly by dysfunctional virus recognition and consecutive defect in response initiation but also to some extent limited responsiveness to autocrine/paracrine IFN.

Normal mammary tissue CD44^+^/CD24^−/low^ stem cells showed a prominent innate immune response to infection with oncolytic virus: 1.6 and 4.8 fold induction of IFNα at 4 h and 24 h time points and 2.5 fold induction of IFNβ at 4 h respectively were detected (Fig. 7A). Interestingly only minor induction of IFNα (1.3 fold) at 4 h and IFNβ (1.4 fold) at 24 h were detected in non-CIC ArLa cells. and no induction of IFNβ at 4 h and IFNα and STAT1 at 24 h time points compared to non-infected cells (Fig. 7A). Similarly, CD44^+^/CD24^−/low^ CIC JIMT-1 cells showed a minor induction of IFNβ (1.2 fold) at 4 h but no induction of IFNα or STAT1. Minor induction of IRF3 (1.6 fold) was detected at 24 h whereas IRF7 could not be detected at this time point (Fig. 7A). In conclusion, the normal mammary tissue CD44^+^/CD24^−/low^ stem cells produced an intact IFN response to virus infection whereas both CD44^+^/CD24^−/low^ CIC and non-CIC population were defective in IFN production when infected with an oncolytic adenovirus.

To verify if the defect in initiation of innate immune responses in JIMT-1 CD44^+^/CD24^−/low^ CIC, is due to dysfunction in responsiveness to autocrine/paracrine type I IFNs in addition to dysfunction in the initial recognition step, we next studied type I IFN response induction by exogenous IFN in JIMT-1 CD44^+^/CD24^−/low^ CIC. The cells were primed with recombinant universal type I interferon prior to infection with Ad5/3-Delta24 or receptor signaling was blocked by interferon receptor neutralizing antibody. IFN induction in pretreated cells in response virus infection was assayed by quantitative real time PCR. Exogenous IFN induced induction of IFNα (13.0 fold), IFNβ (7.2) and STAT1 (7.8 fold) was seen at 4 h (Fig. 7B). However, this induction was rapidly decreased and at 24 h point IFNα, IFNβ and STAT1 levels returned to baseline (Fig. 7B). The exogenous induction of type I IFN was inhibited by treatment with receptor neutralizing antibody (Fig. 7B). In conclusion, JIMT-1 CD44^+^/CD24^−/low^ are able at least to a limited extent to respond to exogenous IFN, however they have a dysfunction in initiation of endogenous type I IFN response upon virus infection.

### STAT activation in type I IFN response regulation upon virus infection

**Figure 8:**
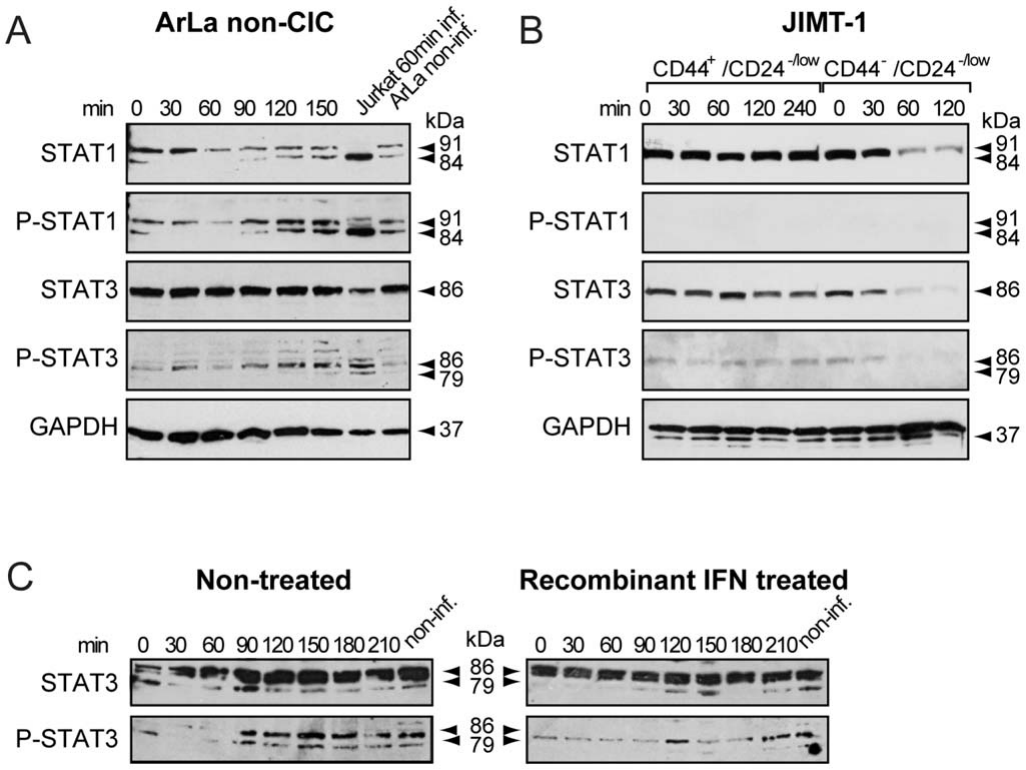
Constant STAT3 and STAT1 activation in non-CIC and no STAT1 activation in CIC. Western blot analysis of STAT1 and STAT3 shows stable upregulation and constant activation in ArLa non-CIC cells with a minor increase in response to infection in STAT1 (A). Jurkat cells infected with Ad5/3-Delta24 are a positive control (A). (B) In JIMT-1 CD44^+^/CD24^−/low^ CIC population there is constant expression of STAT1 however no active P-STAT1 is detected up to 4 h. There is constant expression and activation of STAT3 in CD44^+^/CD24^−/low^ CIC population up to 4 h (B). In contrast in JIMT-1 CD44^−^/CD24^−^ non-CIC population there is decrease in the expression of STAT1 and STAT3 with a decrease also in P-STAT3 (B). ArLa non-CICs do not respond to treatment with exogenous interferon as shown by no changes in STAT3 expression or activation (C).

To explore STAT activation in type I IFN response regulation in ArLa non-CIC cells and JIMT-1 CIC and non-CIC cell populations the cells were sorted and infected with oncolytic adenovirus Ad5/3-Delta24 and STAT1 and STAT3 expression and activation were assayed by Western blot at different time points. ArLa non-CIC cells showed constant activation of STAT1 in infected and non-infected cells (Fig. 8A). There was also constant activation of STAT3 (Fig. 8B). In contrast, in JIMT-1 CD44^+^/CD24^−/low^ CIC and CD44^−^/CD24^−^ non-CIC no active STAT1 was detected (Fig. 8B). In addition, in the JIMT-1 CD44^−^/CD24^−^ non-CIC population expression of both STAT1 and STAT3 decreased 60 min after infection and no more P-STAT3 could be detected at this time point. However, we observed some degree of STAT3 activation at all time points in the CIC population (Fig. 8B). ArLa non-CIC cells treated with recombinant exogenous IFN showed no change in STAT3 expression or phosphorylation status (Fig. 8C).

In conclusion, defects present in ArLa non-CIC and JIMT-1 CIC type I IFN production may be explained by two different mechanisms: In ArLa constitutive STAT activation represses induction of type I IFN response, whereas in JIMT-1 initial defects are present already at the step of virus recognition.

### SOCS and TAM receptors in response to oncolytic adenovirus infection

**Figure 9:**
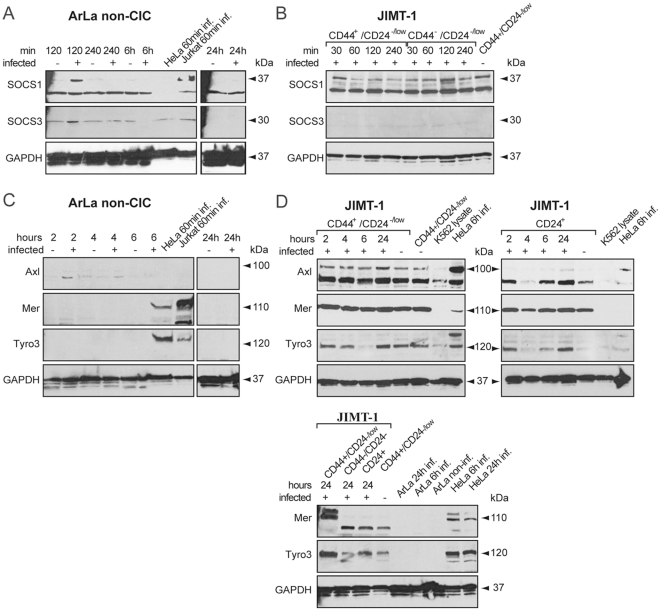
SOCS1, SOCS3 and TAM receptors in CIC and non-CIC. SOCS1, SOCS3 and TAM (Axl, Mer and Tyro3) receptor expression were assayed by Western blot at different time points following infection in non-CIC ArLa cells and JIMT-1 CIC and non-CIC populations. The ArLa non-CIC cells show upregulation of SOCS1 at 2 h and constant expression of SOCS3 with minor upregulation in response to adenovirus infection at 2 h (A). In contrast there is constant expression of SOCS1 in both JIMT-1 CD44^+^/CD24^−/low^ CIC infected and non-infected and infected CD44^−^/CD24^−^ non-CIC populations at all time points (B) and non or minor expression of SOCS3 in both populations (B). No Axl, Mer or Tyro3 induction is seen in any time point in ArLa non-CIC (C). Interestingly, JIMT-1 CD44^+^/CD24^−/low^ CIC show constant upregulation of Axl, Mer and Tyro3 (D) in all time points and also non-infected cells. Infected CD24^+^ non-CIC population show constant Mer and variable Tyro3 expression but no Axl expression (D). Increased inhibitory signaling is likely to contribute to dysfunction in innate immune responses in the CIC population.

To characterize the role of inhibitory SOCS and TAM-receptor signaling in innate immunity regulation, ArLa non-CIC cells and JIMT-1 CIC and non-CIC cell populations were infected with oncolytic adenovirus Ad5/3-Delta24, SOCS1, SOCS3 and Axl, Mer and Tyro3 expression were analyzed by Western blot. The ArLa non-CIC cells showed upregulation of the expression of both SOCS1 and SOCS3 at the 2 h time point but they were no longer detectable at later time points (Fig. 9A). In contrast, there was constant expression of SOCS1 in both infected and non-infected JIMT-1 CD44^+^/CD24^−/low^ CIC infected and non-infected and infected CD44^−^/CD24^−^ non-CIC populations at all time points (Fig. 9B) and no expression or minor expression of SOCS3 in both populations (Fig. 9B).

No Axl, Mer or Tyro3 induction was seen in ArLa non-CIC (Fig. 9C). Interestingly, JIMT-1 CD44^+^/CD24^−/low^ CIC show constant upregulation of Axl, Mer and Tyro3 (Fig. 9D). In infected JIMT-1 non-CIC CD24^+^ population constant Mer expression, but no Axl expression was seen (Fig. 9D).

## Discussion

Increasing evidence suggests that various conventional treatment resistant cancer cell types, including cancer initiating cells, can be targeted by oncolytic viruses. Viruses are not sensitive to drug resistance mechanisms and can overcome defective apoptotic signaling [27]. Our approach utilizes breast cancer patient pleural effusion explant derived cells to model the hierarchical progression of cancer and/or intratumoral heterogeneity. Both ArLa and JIMT-1 represent highly treatment resistant cancer types, the latter featuring also an enrichment of the CD44^+^/CD24^−/low^ containing the CIC cell types. Previous data shows that capsid modified adenoviruses can be used to efficiently target this cell population *in vitro* and *in vivo* [11], [13]. However, as all adult humans have sustained wild type adenovirus infections, normal tissue stem cells must have resistance to viral infection. The mechanisms underlying the differences in sensitivity of CIC *versus* normal stem cells to viral oncolysis have not been studied before.

Various defects in IFN responses confer survival advantages to cancer cells while also resulting in compromised antiviral response. A recent report implicates novel gene sets in oncogenic Ras, TNF and IFN pathways in breast cancer CD44^+^/CD24^−/low^ populations [28]. We show on a functional level that CD44^+^/CD24^−/low^ CIC have defective innate immunity IFN responses while normal mammary tissue cells produce robust responses. Previous reports with other oncolytic viruses show that activation of the interferon pathway protects normal cells while maintaining the vulnerability of cancer cells. For example IFNα or a synthetic inducer of the IFNα/β pathway poly(I:C) completely attenuated oncolytic Vesicular stomatitis virus (VSV) infection of normal brain cells of variable origin, whereas glioblastoma cell lines treated identically were killed by VSV [29]. This indicates that normal stem cells are protected from oncolytic viruses due to intact IFN signaling whereas malignant cells succumb to oncolysis.

PRRs are specialized for sensing pathogen-associated molecular patterns such as viral nucleic acids to induce innate immune responses [30], [31]. Most non-immune cells rely specifically on PPRs to sense infection. Detection via PPRs involves diverse signaling pathways with different cellular outcomes. These can be utilized to circumvent the inherent resistance of cancer cells to current cancer treatments. Inherent defects in specifically the breast cancer CIC population PPRs TLR2 and TLR9 are likely to contribute to dysfunction in the initiation of the type I IFN response leading to a failure of feed-forward signal amplification via IRFs and STAT signaling. This further impacts inhibitory signaling as illustrated by increase of TAM receptors, driven by either inherent upregulation or defects in STAT-SOCS signaling as a compensatory mechanism. On the other hand we also show that while the non-CIC but treatment resistant cells recognize adenovirus by TLR9 and TLR2 have intact PPR signaling *per se*, they still are defective in IFN signaling. This is most probably due to the hyperactivation of STAT3 and/or decreased SOCS expression mediated silencing of cytokine and chemokine production as reported previously in the context of other types of cancer cells [32]. Constitutively activated STAT3 enhances tumor cell proliferation and prevents apoptosis [33]. This also induces the release of factors that inhibit dendritic cell maturation through activation of STAT3 in dendritic cells, negatively regulating induction of adaptive immunity [32].

Non-CIC cancer cells are also unresponsive to exogenous IFN, causing deficiency in paracrine activation. STAT1 is directly suppressed upon adenoviral infection by viral E1A [34]. We used a partly E1A deleted virus, so this interaction may also play a role in downregulation of type I IFN responses even though the CIC population show deficient STAT1 activation. However, constitutive SOCS1 expression and increase in TAM receptor expression in CIC may partly be driven by interaction with viral proteins. Taken together both the CIC and non-CIC conventional treatment resistant populations are defective in type I IFN signaling inducing permissivity to infection. However, this is caused by different mechanisms in each cell population. The mechanisms of viral permissivity in CIC, explored in this study, could further be utilized as prognostic markers, for example looking at primary components or expression levels of downstream targets of the affected pathways.

Strictly controlled cellular localization and trafficking are crucial for the proper function of PPRs in initializing anti-viral responses and avoiding autoimmune reactions. In immune cells TLR9 is expressed at low levels and sequestered in the endoplasmic reticulum (ER) before stimulation. Upon ligand stimulation, it traffics from the ER to the endolysosomes where it binds internalized DNA and initiates a signaling cascade via MyD88 [35], [36], [37]. Moreover, the stimulation results in rapid upregulation of the expression in a positive feedback manner. Interestingly, we show here in normal breast stem cells localization of TLR9 and TLR2 in the sites of recognition, endosomes and cell surface respectively, even without stimulation. This implies constant activation of the receptors and might therefore help explain normal stem cell resistance to wild type virus infection. Moreover, we show for the first time active virus recognition by TLRs in non-immune cell types.

It is possible that, however compromised, the innate immune response might confer resistance against oncolytic viruses to some CIC types. This would partly explain the observed preclinical and clinical findings where tumors were reduced but not completely eradicated or there was a relapse after viral treatment [13]. The presence of highly treatment resistant but differentiated tumor cells could also contribute to this. On the other hand, the type I IFN response induced in the tumor after virus infection could represent an advantage for immunotherapy, where CIC could act as co-adjuvant for the therapeutics.

In summary, this study addresses the mechanisms of innate infection immunity in stem cells, a field which has so far been unexplored. The findings deepen the understanding of stem cell biology and may benefit not only virotherapy but also immunotherapy in general.

## Materials and Methods

### Clinical samples and Cell Culture

This study was conducted according to the principles expressed in the Declaration of Helsinki. The study was approved by the Institutional Review Board of Helsinki University Central Hospital. All patients provided written informed consent for the collection of samples and subsequent analysis.

Cells were isolated from breast tissue derived from reduction mammaplasties. The tissues were dissociated manually and treated with Blenzyme1 collagenase-dispase cocktail 18 h at +37°C strained consecutively through cell strainers, red blood cell lysis was performed and the cells further incubated in Accumax (PAA) to produce single cells. Cells were maintained in MEGM medium with Bullet Kit supplements (Gibco), 1 µl/ml Gentamicin (Sigma) and 1 mg/ml Fungizone/Amphotericin B (Sigma). Cell singularity was confirmed with microscopy and cells were amplified in adherence independent conditions on ultra low-attachment plates (Corning) in reducing amount of FCS to generate spheres.

Cancer cell lines were derived from breast cancer patient pleural effusion derived cells. JIMT-1 (Tanner et al. 2004) are estrogen receptor (ER) and progesterone receptor (PR) negative and human epidermal growth factor receptor 2 (HER-2) positive and ArLa (Courtesy of M. Tanner, Institute of Medical Technology, Tampere University and Tampere University Hospital) ER, PR and HER-2 negative. JIMT-1 were cultured in DMEM/F12 with human recombinant insulin 1 µg/ml (Sigma) 2 mM L-glutamine, 50 U/ml penicillin, 50 µg/ml streptomycin and 10% FCS (Lonza) and ArLa in RPMI-1640 supplemented with antibiotics and 10% FCS (Lonza). For isolation of cell populations the cells were sorted with fluorescein isothiocyanate–labeled anti-CD44 and phycoerythrin-labeled anti-CD24 antibodies (BD Pharmingen, Franklin Lakes, NJ), which were collected with fluorescein isothiocyanate- and phycoerythrin-conjugated magnetic beads, respectively (Miltenyi Biotech, Bergisch Gladbach, Germany). HeLa were maintained in DMEM supplemented with antibiotics and 10% FCS. Jurkat (A3) cells were acquired from ATCC and cultured in RPMI-1640 containing the same supplements.

### Oncolytic adenovirus

The oncolytic adenovirus used in the study is replication competent Ad5/3-Delta24 [38], [39]. It is a serotype 5 based virus retargeted to the adenovirus serotype 3 receptor. The tumor selectivity of Ad5/3-Delta24 is based on a 24 bp deletion in the retinoblastoma (Rb) binding site of E1A [40], [41]. The virus has been shown to infect and kill CD44^+^/CD24^−/low^ cells [11], [13].

### Relative mRNA quantitation by Real-Time PCR

Cells were infected with Ad5/3-Delta24 at multiplicity of infection (moi) 100 VP/cell and total cellular RNA was isolated from cancer cells or mammospheres derived from three pooled donors, using the Qiagen RNeasy kit at different time point. Total cellular RNA (2 µg) was reverse-transcribed into cDNA in TaqMan RT buffer with 1.25 U/µL MultiScribe RT (Applied Biosystems, Foster City, CA, USA). cDNA samples were amplified in Roche universal PCR master mix buffer (Roche) with TaqMan Pre-Developed Assay-on-demand Gene Expression Reagent kits (Applied Biosystems) to analyze mRNA levels for IFN-α1 (Hs00256882_s1), IFNβ1(Hs00277188_s1), IRF3 (Hs00155574_m1), IRF7 (Hs00242190_g1), STAT1 (Hs01014002_m1) and human GAPD (GAPDH) primer limited endogenous control. Each sample was amplified in duplicate or triplicate with a Roche Lightcycler sequence detector (Roche). The relative amounts of cytokine mRNA were calculated with the ΔΔcomparative threshold (Ct) method, and mRNA levels were normalized against GAPDH mRNA. The expression levels of each gene were expressed as fold increase in infected cells at each time point compared to non-treated cells. For exogenous interferon induction cells were in addition pre-treated with IFN-αA/D recombinant universal type I interferon 100 IU/ml (Sigma) and/or anti-human interferon α/β receptor chain2 neutralizing antibody (PBL) 16 h prior to infection.

### Immunofluorescence and Microscopy

Cells were sorted and plated on 6-well plates or coverslips/LabTek chambers (NUNC) respectively and 16 h later infected with adenovirus at the moi 100 VP/cell. 1 h later the medium was changed to growth medium containing 10% FCS. For virus internalization and TLR-receptor studies cells were incubated with virus on ice for 30 min washed with PBS and medium replaced with growth medium containing 10% FCS. Cells were then washed and fixed with 4% PFA 10 min RT and stored at +4°C in PBS. Cells were stained with the following primary antibodies: goat polyclonal anti-adenovirus hexon (ViroStat), mouse monoclonal anti-TLR9 and TLR2 (Invivogen), rabbit polyclonal anti-calnexin, rabbit polyclonal anti-EEA1, (Abcam), mouse monoclonal anti-Oct3/4 (SantaCruz Biotechnology), rabbit polyclonal anti-Musashi, rabbit polyclonal anti-MyD88 (Cell Signaling Technology) and rat monoclonal anti-MyD88 (R&D Systems). Alexa488, Alexa594 and Alexa647 conjugated secondary antibodies were from Molecular Probes/Invitrogen. Cells were mounted with Vectashield with conterstain for nucleai with 4′,6 diamidino-2-phenylindole (DAPI, Vectorlabs). For live cell imaging experiments cells were treated and imaged as described in supplementary materials and methods (Supplementary Materials and Methods S1).

Cells were visualized using Zeiss LSM 5 Duo laser scanning confocal microscope (Jena, Germany). Images were processed for presentation with Adobe Photoshop CS3 and Illustrator CS3 software (Adobe Systems, San Jose, CA).

### Western blot

Cells were seeded at 7.5×10^5^ cells per well on 6-well plates and infected with Ad5/3-Delta24 virus at moi 100. For exogenous IFN induction in ArLa cells human recombinant interferon-αA/D 100 IU/ml (Sigma) was added to the infection medium. After 30 min infection cell were harvested at respective time points in CelLytic M lysis buffer (Sigma). Total protein was measured with Bio-Rad Protein Assay (Bio-Rad) according to the manufacturer's protocol and 50 µg of total protein for each sample were resolved on 4–20% SDS-PAGE gradient gel and transferred onto a nitrocellulose membrane. Membranes were incubated with primary antibodies 1 h at room temperature or overnight at +4°C, followed by incubation with horse radish peroxidase (HRP) conjugated secondary antibodies. Signal detection was performed by enhanced chemiluminescence (Amersham). Primary antibodies: STAT1, phospho-STAT1, STAT3, phospho-STAT3 and SOCS3 (Cell Signaling Technology), GAPDH (Zymed), SOCS1 (Millipore), Axl (Abcam), MerTK (Novus Biologicals), Tyro3 (Santa Cruz Biotechnology) and secondary antibodies anti-rabbit, anti-mouse and anti-goat HRP (Sigma).

## Supporting Information

Figure S1 Defective trafficking of TLR9 and MyD88 in JIMT-1 CD44^+^/CD24^−/low^ CIC but not ArLa non-CIC. To further investigate whether the localization of TLR9 in the JIMT-1 CD44^+^/CD24^−/low^ CIC population was related to the trafficking of the protein, we followed TLR9 and MyD88 in JIMT-1 CD44^+^/CD24^−/low^ CIC and ArLa non-CIC upon transient transfection of constructs expressing fluorescently labeled proteins and organelle markers and live cell imaging. Cells were sorted, transfected, and infected the following day. Cells were then treated with cycloheximide to stop protein synthesis and the fluorescently labeled proteins were followed by live cell imaging for up to 6 hours. In JIMT-1 CD44^+^/CD24^−/low^ CIC, at 4 h after infection with Ad5/3-Delta24, transfected TLR9-YFP showed retention in the ER-Golgi similarly to the endogenous protein (Fig. S1A). Similarly the transfected MyD88-CFP, a cofactor of TLR signaling, was also retained in the ER-Golgi and did not localize in endosomes in JIMT-1 CD44^+^/CD24^−/low^ CIC (Fig. S1B). In ArLa non-CIC transfected TLR9-YFP and MyD88-CFP travel through the Golgi at 1 h time point after infection (Fig. S1C) and reach the endosomes at four hours after infection (Fig. S1D).
(5.48 MB TIF)

Materials and Methods S1 (0.01 MB DOCX)
