# Supplementary figures and images for: Defects in Innate Immunity Render Breast Cancer Initiating Cells Permissive to Oncolytic Adenovirus
Source: PLoS One. 2010 Nov 5;5(11):e13859. doi: 10.1371/journal.pone.0013859 (PMC2974645; doi:10.1371/journal.pone.0013859)

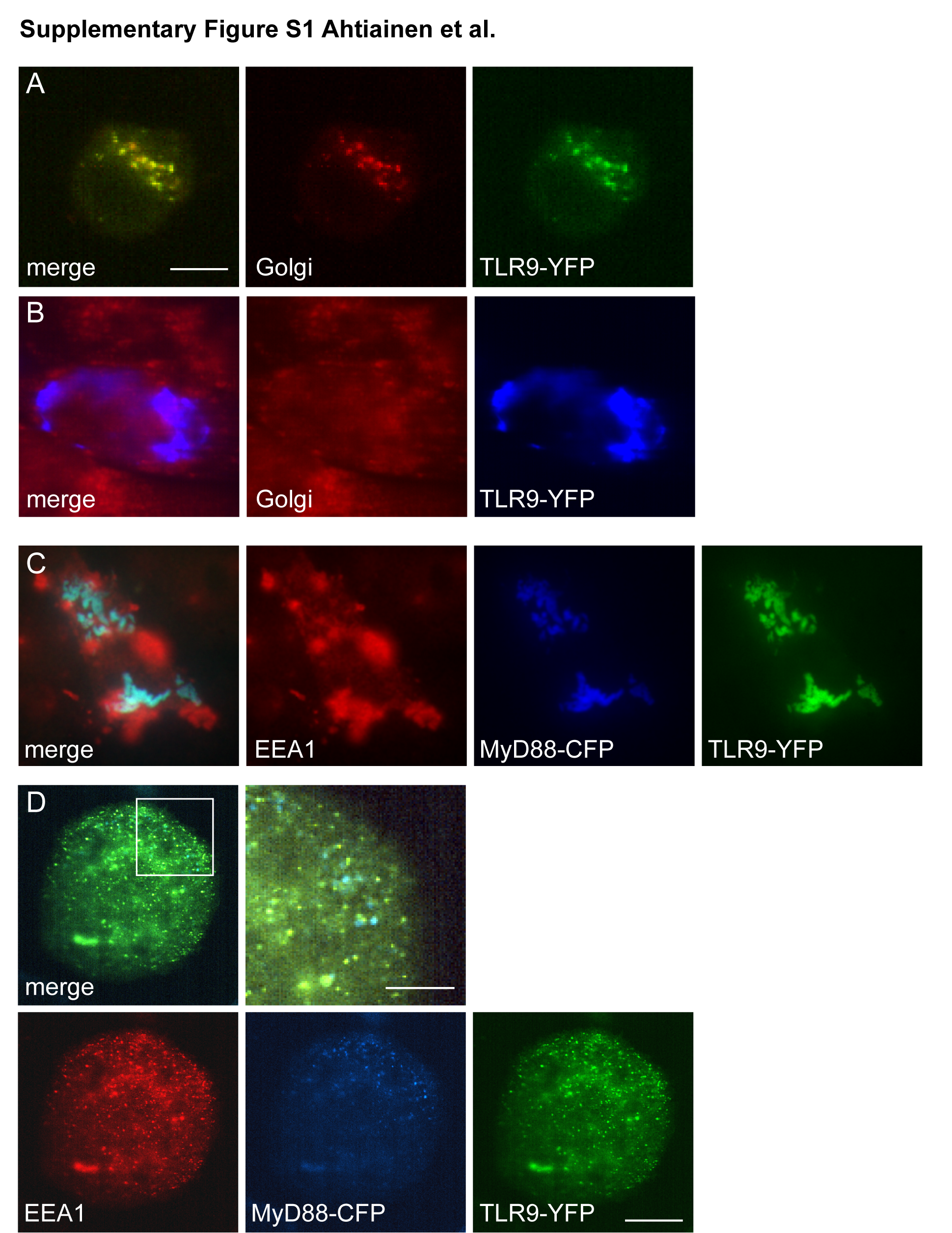

Supplement: Figure S1 — Defective trafficking of TLR9 and MyD88 in JIMT-1 CD44+/CD24−/low CIC but not ArLa non-CIC. To further investigate whether the localization of TLR9 in the JIMT-1 CD44+/CD24−/low CIC population was related to the trafficking of the protein, we followed TLR9 and MyD88 in JIMT-1 CD44+/CD24−/low CIC and ArLa non-CIC upon transient transfection of constructs expressing fluorescently labeled proteins and organelle markers and live cell imaging. Cells were sorted, transfected, and infected the following day. Cells were then treated with cycloheximide to stop protein synthesis and the fluorescently labeled proteins were followed by live cell imaging for up to 6 hours. In JIMT-1 CD44+/CD24−/low CIC, at 4 h after infection with Ad5/3-Delta24, transfected TLR9-YFP showed retention in the ER-Golgi similarly to the endogenous protein (Fig. S1A). Similarly the transfected MyD88-CFP, a cofactor of TLR signaling, was also retained in the ER-Golgi and did not localize in endosomes in JIMT-1 CD44+/CD24−/low CIC (Fig. S1B). In ArLa non-CIC transfected TLR9-YFP and MyD88-CFP travel through the Golgi at 1 h time point after infection (Fig. S1C) and reach the endosomes at four hours after infection (Fig. S1D). (5.48 MB TIF) [file pone.0013859.s001.tif]
