# Supplementary material for: Defects in Innate Immunity Render Breast Cancer Initiating Cells Permissive to Oncolytic Adenovirus
Source: PLoS One. 2010 Nov 5;5(11):e13859. doi: 10.1371/journal.pone.0013859 (PMC2974645; doi:10.1371/journal.pone.0013859)
Supplement: Materials and Methods S1 — (0.01 MB DOCX) [file pone.0013859.s002.docx]

**Supplementary Materials and Methods**

pcDNA3-TLR9-CFP (Addgene plasmid 13642), pcDNA3-MyD88-YFP (Addgene plasmid 13026), pcDNA3-CFP (Addgene plasmid 13030) and pcDNA3-YFP (Addgene plasmid 13033) were acquired from Addgen/Dr D. Golenbock, Division of Infectious Diseases and Immunology, University of Massachusetts Medical School.

Cells were sorted and plated on LabTek chambers (NUNC). Cells were transfected with fluorescently tagged receptor expressing constructs 24h prior to infection with Effectene transfection reagent (Qiagen) and BacMam expression and delivery reagents for fluorescent organelle markers: Organelle Lights Endosomes-Red, Organelle Lights Golgi-Red (Molecular Probes/Invitrogen). Cells were infected with adenovirus at the multiplicity of infection 100 VP/cell. 1h later the medium was changed to growth medium containing 10% FCS and supplemented with 10mM HEPES and 100µg/ml cycloheximide 1h before visualization.

Cells were imaged with Zeiss LSM 5 Duo laser scanning confocal microscope with LSM 5 Live line-scanner fitted with inverted Zeiss Axio Observer Z1 microscope with an environmental chamber. Images were processed for presentation with Adobe Photoshop CS3 and Illustrator CS3 software (Adobe Systems, San Jose, CA).
